# Effects of a free school breakfast programme on school attendance, achievement, psychosocial function, and nutrition: a stepped wedge cluster randomised trial

**DOI:** 10.1186/1471-2458-10-738

**Published:** 2010-11-29

**Authors:** Cliona Ni Mhurchu, Maria Turley, Delvina Gorton, Yannan Jiang, Jo Michie, Ralph Maddison, John Hattie

**Affiliations:** 1 Clinical Trials Research Unit, School of Population Health, Faculty of Medicine & Health Sciences, The University of Auckland, Private Bag 92019, Auckland 1142, New Zealand; 2 Teaching, Learning and Development, Faculty of Education, The University of Auckland, Private Bag 92019, Auckland 1142, New Zealand

## Abstract

**Background:**

Approximately 55,000 children in New Zealand do not eat breakfast on any given day. Regular breakfast skipping has been associated with poor diets, higher body mass index, and adverse effects on children's behaviour and academic performance. Research suggests that regular breakfast consumption can improve academic performance, nutrition and behaviour. This paper describes the protocol for a stepped wedge cluster randomised trial of a free school breakfast programme. The aim of the trial is to determine the effects of the breakfast intervention on school attendance, achievement, psychosocial function, dietary habits and food security.

**Methods/Design:**

Sixteen primary schools in the North Island of New Zealand will be randomised in a sequential stepped wedge design to a free before-school breakfast programme consisting of non-sugar coated breakfast cereal, milk products, and/or toast and spreads. Four hundred children aged 5-13 years (approximately 25 per school) will be recruited. Data collection will be undertaken once each school term over the 2010 school year (February to December). The primary trial outcome is school attendance, defined as the proportion of students achieving an attendance rate of 95% or higher. Secondary outcomes are academic achievement (literacy, numeracy, self-reported grades), sense of belonging at school, psychosocial function, dietary habits, and food security. A concurrent process evaluation seeks information on parents', schools' and providers' perspectives of the breakfast programme.

**Discussion:**

This randomised controlled trial will provide robust evidence of the effects of a school breakfast programme on students' attendance, achievement and nutrition. Furthermore the study provides an excellent example of the feasibility and value of the stepped wedge trial design in evaluating pragmatic public health intervention programmes.

**Trial Registration Number:**

Australian New Zealand Clinical Trials Registry (ANZCTR) - ACTRN12609000854235

## Background

Up to one-fifth of New Zealand children leave for school without eating breakfast [1-3]. Missing breakfast is more common amongst older children, girls, Māori and Pacific children, and those living in lower socioeconomically resourced areas [2,3]. Missing breakfast has been associated with adverse effects on cognitive function (including memory), academic performance, school attendance, psychosocial function and mood in children and young people [4,5]. Conversely, breakfast consumption is associated with a range of positive outcomes, including better school attendance, academic performance, nutrient intake, fitness, and healthier body weight [6-8].

Breakfast contributes substantially to daily energy and nutrient intake. Breakfast provides 16% of New Zealand children's daily energy intake, around one-third of calcium, iron, thiamine, riboflavin and folate intakes, and one-fifth of zinc intake [9]. Children who miss breakfast have significantly worse daily nutrient intakes, including higher intakes of total fat, and lower intakes of dietary fibre and micronutrients than those who eat breakfast [9]. Hungry children may lack the energy and motivation to become involved in classroom activities [10], while malnutrition and micronutrient deficiencies have been shown to impact on physical, mental, and social health, and reduce cognitive functioning [11-13].

A number of countries (e.g. United States, United Kingdom, Sweden) have government or non-government funded school breakfast programmes, which aim to provide a free healthy breakfast to children and thereby improve nutrition and academic outcomes [14-16]. In New Zealand, some school breakfast programmes have been introduced in recent years, although none are official government programmes. The Red Cross Breakfast in Schools programme, which is available to decile 1 primary schools (areas of low socioeconomic resource), was established in 2007 [17]. The programme is run with support from Countdown supermarkets (Progressive Enterprises) who supply breakfast foods free of charge. In 2008, the KickStart Breakfast programme for decile 1-4 schools (areas of low to moderate socioeconomic resource) was launched by Fonterra Co-Operative Group Limited and Sanitarium Health Food Company.

Research indicates that school breakfast programmes have benefits in relation to nutrition, school attendance, academic performance and psychosocial function [14,18]. However, findings have been inconsistent, limited by poor study design and methods, and frequently confuse correlation with causation. A Cochrane review identified seven randomised, controlled trials investigating the effects of school feeding programmes (breakfast, lunch or snacks) [18]. However, only two were undertaken in high income countries, one of which experienced substantial contamination between trial intervention groups [19], whilst the other did not include a full school breakfast (just milk) [20]. Based on all studies, the Cochrane review concluded that there is a dearth of high quality evidence on school feeding programmes and recommended further well-designed studies, particularly cluster randomised controlled trials [18].

This paper presents the study protocol for a stepped wedge cluster randomised controlled trial to investigate the effects of a free school breakfast programme on students' school attendance, academic achievement, psychosocial function, dietary habits and food security.

## Method/Design

In New Zealand, there are two stages of schooling: primary (years 0-8) and secondary (years 9-15). Primary schools include full primary (years 0-8), contributing (years 0-6) and intermediate (years 7-8) schools. There are four terms in a primary school year, with term 1 starting in early February and term 4 ending in December. All schools are assigned a decile rating, which indicates the extent to which the school draws its students from a range of socioeconomic areas. Decile 1 schools are the 10% of schools with the highest proportion of students from low socioeconomic resource areas (defined according to residents' income, occupation, household crowding, educational qualifications and income support) and decile 10 are the 10% of schools with the highest proportion of students from high socioeconomic areas.

### Design

**Figure 1:**
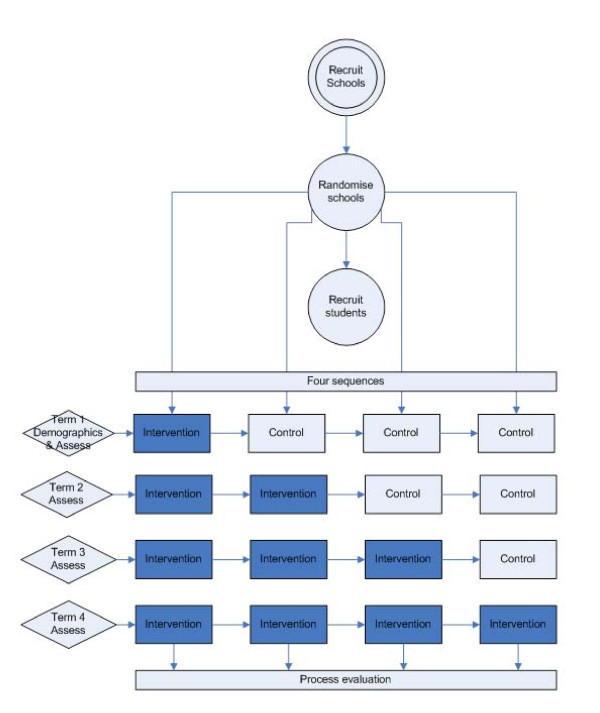
**Stepped wedge cluster randomised trial design**.

The study is a stepped wedge cluster randomised controlled trial in which participating schools (clusters) cross over from control to intervention phase (i.e. one-way switch over) at different time points throughout the school year. The order of switch-over ('sequence') is determined randomly for each group of clusters, and all clusters receive the breakfast programme intervention by the end of study (Figure 1). All schools start the trial at the same time point and act as controls until such time as they are randomised to crossover from control to intervention. The stepped wedge design is suitable for a phased evaluation approach, where the intervention is likely to do more good than harm, thus making it unethical to completely withhold the intervention from a control group, and/or where it is not possible to start delivery of the intervention to all participants at the same time [21]. Both of these criteria apply in this trial. Data on all individual study participants are collected once each school term over the entire school year.

### Aims

The primary aim is to investigate the effects of a school breakfast programme in low decile schools on students' attendance. Secondary aims include examination of the impact of the school breakfast programme on students' school achievement, psychosocial function and nutrition.

### Study sample

Eligible schools are decile 1-4 primary schools (students aged 5-13 years) located in selected regions (Auckland, Northland, Waikato, Wellington) in the North Island of New Zealand. To take part in the study, schools must not have an existing breakfast programme and must agree to start one in the 2010 school year. Schools must be of sufficient size that they estimate at least 25 students would attend the breakfast programme each day.

Eligible participants are students aged 5-13 years who plan to attend the school breakfast club at least occasionally and do not have a diagnosed food allergy. Only one child per household may take part. Recruitment involves researchers and/or the school principal briefly outlining the study during a regular school assembly or classroom talks and answering any questions children have. Those interested in taking part in the study are asked to register their interest and take a study information pack home to their parents/caregivers. The study pack includes participant information, a consent form for parents, an assent form for children, forms to collect demographic information (e.g., age, sex and ethnicity of the child, household income) and parents' contact details, and an envelope in which to return completed forms. Students return completed forms to their teacher or the school office, and these are collected by researchers.

### Sample size calculations

The target sample size is a total of 16 schools (four schools per sequence) with an average 25 students per school, i.e. a total of 400. Assuming an intra-cluster coefficient (ICC) of 0.05, this sample size will provide at least 85% power, with a significance level of α = 0.05, to detect a 10% absolute increase in the proportion of students with a school attendance rate of 95% or higher.

### Ethics approval

Ethical approval for the trial was received from the Northern Y Regional Ethics Committee (Reference: NTY/09/09/084).

### Randomisation and blinding

Schools are randomly allocated to one of the four sequences for time of crossover from control to intervention phase using a computer-generated list of random numbers. The allocation sequence is overseen by the study statistician (YJ). Consideration is given to school decile and school type in order to reduce the potential imbalance between sequences. Due to the nature of the study intervention it is not possible to blind participants, breakfast providers, or outcome assessors. However, choice of an objective primary outcome (school attendance using routinely collected data from schools) should minimise risk of bias.

### Intervention

The intervention is a free daily before-school breakfast programme; either the Red Cross Breakfast in Schools programme or an unbranded school breakfast programme provided by Fonterra and Sanitarium.

The Red Cross Breakfast in Schools programme is available to decile 1 primary schools. Food is provided free of charge by Countdown supermarkets (Progressive Enterprises Ltd). Schools select from a list of foods that includes breakfast cereal (wheat biscuits), milk, bread, spreads (margarine, jam, honey, Marmite), Milo (chocolate flavoured drink powder), milk powder and sugar. Food is delivered weekly. During the trial the programme is set up and run as usual through the Red Cross, with schools responsible for finding volunteers to run the programme and Red Cross providing assistance and training. The unbranded school breakfast programme is available to decile 1-4 primary and intermediate schools. Food is provided by Fonterra Co-operative Group Limited (fresh or UHT milk) and Sanitarium Health Food Company (Weet-Bix). Milk is provided weekly (where fresh milk is supplied) and Weet-Bix and UHT milk delivered each term. During the trial the programme is set up and run as usual, with schools providing volunteers to run the breakfast programme.

Schools are provided with information on how to set up and run a breakfast club, but are responsible for finding a suitable venue, equipment and volunteers to run the breakfast club. Schools are offered NZ$1000 as reimbursement for their participation in the trial, and this may be used to help set up the breakfast club or for other purposes. No attempts are made to standardise the intervention or its delivery in individual schools.

### Study outcome measures

Assessments (Table 1) are undertaken before school starts to minimise disruption to students' schooling. For schools that have started the intervention, assessments take place at the breakfast club venue after students have eaten breakfast and before they start school. In control schools assessments take place at a suitable venue, such as a hall or classroom. Students complete questionnaires independently where possible. Questions are read to younger students or those who have difficulty understanding.

**Table 1:** Primary and secondary outcome measures

Outcome	Instrument/Method	Collected from
Attendance	Recorded school attendance	Schools
Academic achievement	Literacy and numeracy	Schools
Self-reported grades	Study questionnaire [24]	Students
Sense of belonging	PISA 2000 Sense of Engagement Scale [25]	Students (Year 4 and above)
Psychosocial function	Strengths and Difficulties Questionnaire [26,27]	Teachers
Hunger	"Freddy" [28]	Students
Dietary habits	Dietary Habits Questionnaire (breakfast only) [29,2]	Parents
Food security	CCHIP scale [30,31]	Parents

#### Primary outcome

Attendance is defined as total number of half days students are present at school (including those slightly late for the morning or afternoon session e.g. due to a medical appointment). The main measure of attendance is the proportion of students achieving a school attendance rate of 95% or higher, which equates to students missing less than 2-3 days per school term. Other attendance outcomes are number of late, sick or medical days, justified absences (e.g. bereavement leave), and unjustified absences (truancy). Attendance data are recorded by schools in the usual way and extracted for data collection at the end of each school term.

#### Secondary outcomes

Academic achievement is assessed using numeracy and literacy data collected by schools using standardised tests administered to all students during the year. New Zealand schools use a limited range of tests to assess numeracy and literacy (e.g., asTTle, PAT, SEA), usually during terms 1 and 4. Data will be collated, integrated, and analysed by an expert in academic assessment (JH), taking into account any maturation effect, thus permitting assessment on the same scale.

Self-reported grades (i.e., children's perceived academic competence) are a determinant of actual academic performance in core subjects [22,23]. Perceived academic competence is measured by asking students to make a realistic assessment of their reading ability in comparison to other students in their year at school, with five response options ranging from 1 (not very well) to 5 (very well) [24].

Students' sense of belonging is measured using the PISA 2000 Student Engagement Questionnaire, which consists of six statements with four response options ranging from strongly disagree to strongly agree [25]. It measures whether students feel comfortable and as if they belong at school, as well as their relationship with other students. Although designed for use with 15 year-old students, it has a readability level appropriate for younger students. Only students in Year 4 or above (approximately 8 years or older) complete this questionnaire.

The Strengths and Difficulties Questionnaire (SDQ) [26,27] assesses students' behaviour, emotions and relationships and is completed by the student's teacher. The SDQ consists of 25 items related to five dimensions: hyperactivity/inattention, emotional symptoms, conduct problems, peer relationship problems, and prosocial behaviour.

Temporary hunger levels are assessed using a modified version of "Freddy", an analogue scale (1-15) for measuring fullness or satiety in children [28]. Freddy has been validated for use in the United States and was pre-tested with decile 1 New Zealand primary schoolchildren aged 6-11 years (n = 21) prior to the start of the study. Pre-testing demonstrated expected increases in mean satiety ratings between beginning, middle and completion of breakfast. Furthermore the instrument showed good test-retest reliability: median scores at the beginning of breakfast were 6.4 on day 1 and 5.9 on day 2, whilst median scores at the end were 14.5 and 14.4 respectively. There were no significant differences in scores between the two days (p-values > 0.7).

Students' dietary habits over the past week are assessed using questions from previous national surveys [29,2]. Three questions assess how often students eat breakfast, where they eat breakfast, and where they source food for breakfast.

The CCHIP scale [30,31] is used to assess food security status. CCHIP was chosen because this instrument differentiates between household and child food security, whereas most other measures focus on household food security. The scale consists of eight questions, two about the household, two about adults in the household, and four about the child/children in the household.

### Process evaluation

A concurrent process evaluation will collect information from school breakfast providers, schools, and parents on their perceptions of the breakfast programme and any challenges associated with its implementation and sustainability. School and parent evaluations will be undertaken at the end of the study using standardised questionnaires. Evaluation of breakfast provider perspectives will be undertaken via semi-structured interviews on completion of the trial.

### Statistical analysis

Treatment evaluations will be performed according to the principle of intention-to-treat (ITT), using data collected from individual trial participants at randomised schools. Schools will be treated as clusters in the analysis because participant data collected from the same school are likely to be more correlated than those collected from different schools. Furthermore, in a stepped wedge design, information is collected repeatedly on each participant over the control and intervention periods. Advanced statistical methods appropriate for this type of study design will therefore be used. Generalised Linear Mixed models will be used to adjust for the effect of clustering and repeated measures over time. Intra cluster correlation (ICC) coefficients will be re-evaluated using the study data. Unidirectional crossover as well as any potential secular trends may be incorporated with a time effect. A pre-specified analysis plan outlines detailed statistical methods.

## Discussion

The breakfast intervention trial described in this paper is the first of its kind in Australasia and amongst only a very small number conducted in high income countries to date. Whilst evidence suggests that school breakfast programmes have beneficial effects on student attendance, school grades, and psychosocial function, findings to date have been inconsistent and limited by poor study methodology. This study addresses many of the shortfalls in current research into the effects of school breakfast programmes by employing a prospective cluster randomised trial design to minimise confounding and bias, thus substantially increasing confidence in findings.

Results from this research will provide valuable, much needed information on the effects of free school breakfast programmes in high income countries on school attendance, achievement, psychosocial function, and nutrition.

Furthermore this trial provides an excellent example of the feasibility and value of the stepped wedge trial design in evaluation of pragmatic public health interventions. All too often such initiatives do not undergo robust evaluation due to difficulties in aligning the implementation timetable and rollout strategy with trial design and recruitment [32]. This trial provides a useful illustration of simultaneous implementation and rigorous evaluation of a public health intervention programme.

## Competing interests

The authors declare that they have no competing interests.

## Authors' contributions

Principal responsibility for study design was assumed by DG, YJ, RM and CNM. DG, JM and MT developed the study protocol and materials. JH had substantial input into study design and oversight. JM is responsible for day-to-day study management. YJ is responsible for all aspects of statistical design and analysis. CNM drafted the study manuscript integrating substantial written contributions from MT. All authors contributed to and approved the final manuscript.

## Pre-publication history

The pre-publication history for this paper can be accessed here:

http://www.biomedcentral.com/1471-2458/10/738/prepub
